# HIV among Female Sex Workers in Five Cities in Burkina Faso: A Cross-Sectional Baseline Survey to Inform HIV/AIDS Programs

**DOI:** 10.1155/2017/9580548

**Published:** 2017-11-15

**Authors:** Henri Gautier Ouedraogo, Odette Ky-Zerbo, Adama Baguiya, Ashley Grosso, Sara Goodman, Benoît Cesaire Samadoulougou, Marcel Lougue, Nongoba Sawadogo, Yves Traore, Nicolas Barro, Stefan Baral, Seni Kouanda

**Affiliations:** ^1^Institut de Recherche en Sciences de la Santé (IRSS), Ouagadougou, West Africa, Burkina Faso; ^2^University Ouaga 1 Joseph Ki-Zerbo, Ouagadougou, West Africa, Burkina Faso; ^3^Institut Africain de Santé Publique, Ouagadougou, West Africa, Burkina Faso; ^4^Programme d'Appui au Monde Associatif et Communautaire, Ouagadougou, West Africa, Burkina Faso; ^5^Department of Epidemiology, Johns Hopkins Bloomberg School of Public Health, Baltimore, MD, USA; ^6^Centre Hospitalier Regional de Kaya, West Africa, Burkina Faso

## Abstract

**Background:**

Female sex workers (FSWs) are considered a vulnerable population for HIV infection and a priority for HIV/AIDS response programs. This study aimed to determine HIV prevalence among FSWs in five cities in Burkina Faso.

**Methods:**

FSWs aged 18 and older were recruited using respondent driven sampling (RDS) in five cities (Ouagadougou, Bobo-Dioulasso, Koudougou, Ouahigouya, and Tenkodogo) in Burkina Faso from 2013 to 2014. HIV testing was performed using the HIV testing national algorithm. We conducted bivariate and multivariate logistic regression analysis to assess correlates of HIV in all cities combined (not RDS-adjusted).

**Results:**

Among Ouagadougou, Koudougou, and Ouahigouya FSWs, RDS-adjusted HIV prevalence was 13.5% (95% Confidence Interval [CI]: 9.6–18.7), 13.3% (95% CI: 7.6–22.4), and 13.0% (95% CI: 7.6–21.3), respectively, compared to 30.1% (95% CI: 25.5–35.1) among Bobo-Dioulasso FSWs. Factors associated with HIV infection were age (adjusted odds ratio [aOR] = 7.84 95% CI: 3.78–16.20), being married or cohabitating (aOR = 2.43, 95% CI: 1.31–4.49), and history of pregnancy (aOR = 5.24, 95% CI: 1.44–18.97).

**Conclusion:**

These results highlight the need to strengthen HIV prevention among FSWs, through behavior change strategies, and improve access to sexual and reproductive health services.

## 1. Introduction

The HIV epidemic is still a major concern in low- and middle-income countries, specifically in sub-Saharan Africa [1]. The World Health Organization (WHO) African Region is the most affected globally, with 25.6 million people living with HIV out of the 36.7 million people living with HIV worldwide at the end of 2016 [2]. Although the global burden of HIV has significantly decreased, in West Africa, the epidemic is concentrated mainly in specific groups and vulnerable populations including female sex workers (FSWs) [3, 4]. Female sex workers are defined as women aged 18 and older who sell consensual sexual services in return for cash or payment in kind and who may sell sex formally or informally, regularly or occasionally [5]. Compared to women not practicing sex work, FSWs are at increased vulnerability to contract HIV and sexually transmitted infections (STIs) [5–12].

A review of FSW studies in low- to middle-income countries indicated that FSWs in sub-Saharan Africa had more than 12 times the risk of contracting HIV compared with all women of reproductive age [13]. Studies conducted in Swaziland (70.4%) and Uganda (37%) among FSWs showed higher HIV prevalence [14, 15]. Papworth et al. reported, in a systematic review on HIV among FSWs, their clients, men who have sex with men, and people who inject drugs in 24 countries of Central and Western Africa, an overall HIV prevalence of 34.9% among FSWs with differences across countries [8]. HIV prevalence among FSWs varies within and across African settings [8, 16]. In West and Central Africa, epidemiological data on vulnerable populations remain either outdated or partial and are mostly from the largest cities with no data from smaller cities. Several studies largely conducted in urban areas in West and Central Africa have reported HIV prevalence among FSWs to be 15.9% in Gambia, 20.0% in Nigeria, 45.4% in Togo, 68.6% in Ghana and Benin, and 58.2% in Burkina Faso [14–18].

In Burkina Faso, the Joint United Nations Programme on HIV/AIDS (UNAIDS) consecutive reports on the HIV epidemic show a decline in HIV prevalence in the overall population of Burkina Faso over time, from 2.7% in 2003 to 1.0% in 2010 and then to 0.9% in late 2014 [19]. Although the HIV prevalence in Burkina Faso among people of reproductive age (ages 15 to 49) is low, the epidemic is concentrated in vulnerable groups such as FSWs [20]. One study reported that HIV prevalence was 6.5% among part-time sex workers in Burkina Faso and 10.3% among full-time sex workers, much higher than the national rates for people of reproductive age [20]. However, this study was limited to FSWs aged 18 to 25 in Ouagadougou alone. National and international programs addressing HIV in regard to FSWs lack basic information regarding this population which impedes evidence-based program planning, implementation, monitoring, and evaluation. This study aimed to determine HIV prevalence and correlates of HIV infection among FSWs in two large and three small cities in Burkina Faso.

## 2. Materials and Methods

### 2.1. Study Design

We conducted a cross-sectional biological and behavioral study among FSWs in Burkina Faso, using respondent driven sampling (RDS) [21, 22]. Eligibility criteria included the following: (1) being at least 18 years old, (2) assigned female sex at birth, (3) having at least 50% of annual income from sex work in the past 12 months, (4) having stayed in the city at least for the past three months, (5) having a valid study coupon, and (6) being able to provide informed consent for participation in study activities.

### 2.2. Setting

We selected the two largest cities: the capital, Ouagadougou in the Central region, and Bobo-Dioulasso in the Hauts-Bassins region (western Burkina Faso), and three smaller cities: Koudougou in the West Central region, Ouahigouya in the North region, and Tenkodogo in the East Central region. These cities were selected for their level of urbanization, HIV prevalence, and geographic location. Formative research with FSWs, local FSW organizations, and government officials took place using a formal meeting in each site to inform the study procedures. Data were collected from February 2013 to May 2014.

### 2.3. Study Size

The sample size was calculated to recruit 345 FSWs in the two largest cities (Ouagadougou and Bobo-Dioulasso) and 126 FSWs in the smaller ones (Koudougou, Ouahigouya, and Tenkodogo). Sample size calculations were based on the assumption that populations that always use condoms have a 75% lower HIV prevalence than populations who do not, and the effectiveness of condoms is roughly 80%, with 73% as a conservative estimate [23]. Overall, across all cities, HIV prevalence was estimated at 15%, with a 19% prevalence among those who did not consistently use condoms [24, 25]. A design effect of 1.5 associated with RDS and significance level of 0.05 and a power of 80% were employed.

### 2.4. Study Participants

Respondent driven sampling, a peer-driven sampling method designed to reach hidden populations such as FSWs [21, 26], was selected in order to collect rigorous, representative data. Respondent driven sampling starts with eligible “seeds” to start recruitment chains [21, 26]. For each site, three to ten seeds were selected based on diverse sociodemographic selection criteria, including popularity, sociability, age, location, type of sex work, and nationality, with the assumption that each individual represented a different social network within the FSW population as a whole in each study site. After completing study procedures, these seeds were each provided with three coded coupons, valid for four weeks, to recruit peer FSWs from their social network. This process continued until the desired sample size was reached.

All participants received male condoms, condom-compatible lubricants, HIV education materials, and information regarding existing services. They also received 2000 West African CFA franc (XOF) (United States [US] $4) for their time and transportation costs for each study visit and 1500 XOF (US$3) per successfully recruited eligible peer (for up to three peers). To avoid individuals participating multiple times, a single study office was used in each study site in addition to the use of a unique identification code. Trained staff at each site included a site manager, a coupon manager, two data collectors, an HIV test counsellor, and a lab technician. Full details of the study methodology have been previously described [27].

### 2.5. Data Collection

Data were collected from February 2013 to May 2014. After informed consent, each participant completed a private interviewer-administered questionnaire in French or the local language. Topics included demographic and socioeconomic characteristics, sexual partnerships and behaviors (including condom use during the last 12 months and condom use at last sex with a new client), and knowledge, attitudes, and practices related to STIs and HIV based on the modified social ecological model [28]. After the questionnaire, pre- and posttest HIV counselling, based on the standard national counselling protocol, was conducted. A venous blood specimen (~5 milliliters) was collected from each consenting participant for HIV testing.

### 2.6. HIV Testing

HIV testing was performed using the national Burkina Faso HIV testing algorithm (Presidency of Burkina Faso/CNLS: HIV testing and diagnosis algorithm in Burkina Faso. Oct. 2004.). The first step was to perform a rapid test using Alere Determine™ HIV-1/2 kit (Alere, Inc., Waltham, Massachusetts). This was followed by ImmunoComb® II HIV 1&2 BiSpot kit (Alere, Inc.) as a second test for differential detection of antibodies to HIV types 1 and 2, only if the first test was positive. Four discordant results were further tested and confirmed negative through use of the ImmunoComb II HIV 1&2 CombFirm kit (Alere, Inc.).

### 2.7. Data Processing and Analysis

Data were entered using double data entry into EpiData 3.1 (The EpiData Association, Odense, Denmark) and exported into Stata 14 (StataCorp, College Station, TX) for analysis. Descriptive statistics were used to describe participants' characteristics, sexual behaviors, condom use, and HIV prevalence. We adjusted all proportions separately for each city to account for the RDS method. This adjustment takes into consideration the probability of each participant to be included in the study. This probability was measured through weighting based on the size of each participant's network. Network size was determined using the survey question: “how many different people do you know personally who are female sex workers or sell sex? i.e., you know them and they know you, and you could contact them if you needed to?” The mean network size was 39: the network size by city was 69 in Ouagadougou, 21 in Bobo-Dioulasso, 39 in Koudougou, 13 in Ouahigouya, and 27 in Tenkodogo. Network size ranged from 1 to 1000. We presented population estimates and 95% confidence intervals (CI) adjusted for RDS design using the RDS Analysis Tools (RDSAT) version 6.0.1 (RDS, Inc., Ithaca, NY). Bivariate and multivariate logistic regression analyses were performed using Stata to identify factors associated with HIV infection at the *p* < 0.05 level of significance along with their 95% confidence interval (CI). These pooled bivariate and multivariate analyses were not RDS-adjusted because data from all cities were combined. Multivariate analyses were not conducted separately for each city due to smaller sample sizes in Koudougou, Ouahigouya, and Tenkodogo. Age categories were generated according to existing HIV planning goals with adolescent FSWs categorized as age 24 and younger.

Our outcome variable was HIV status (positive or negative) as determined by blood tests. Predictor variables included sociodemographic variables including age, education level, marital status, employment, and migration to Burkina Faso. Other predictor variables included those related to sex work including experience, number of clients, and condom use. First, sociodemographic and behavioral variables associated with HIV infection at the significance level of *p* < 0.2 in bivariate analyses were included in a backward elimination model selection procedure, and variables independently associated with HIV infection were retained in the multivariate model to produce the final results.

## 3. Results

### 3.1. Descriptive Statistics

As shown in Table 1, overall, 1073 FSWs (349 in Ouagadougou, 350 in Bobo-Dioulasso, 117 in Koudougou, 121 in Ouahigouya and 136 in Tenkodogo) were included in this study. The mean age of participants varied across cities, from 23.9 (±5.1) years in Tenkodogo to 30.7 (±8.6) years in Bobo-Dioulasso.

#### 3.1.1. RDS-Adjusted Descriptive Statistics

*Sociodemographic Characteristics of FSWs*. Participants' education level was low in general with many who never attended school. Almost one-third of those of Ouagadougou (31.6%, 95% CI: 26.5–37.2) and in Koudougou (32.3%, 95% CI: 24.0–41.9) as well as half (49.2%, 95% CI: 43.8–54.5) of those of Bobo-Dioulasso had never been to school. The majority of FSWs in each city had no other job besides sex work. In terms of marital status, more than half of FSWs were single ranging from the lowest frequency of 52% (95% CI: 46.6–57.1) in Bobo-Dioulasso to 74% (95% CI: 65.2–81.2) in Tenkodogo. More than a third of them were divorced, separated, or widowed in Ouagadougou and Bobo-Dioulasso, whereas, in Koudougou, Ouahigouya, and Tenkodogo, this group represented less than a quarter of participants.

More than half of FSWs in Tenkodogo (59.8%, 95% CI: 44.7–55.4) and Ouagadougou (50.0%, 95% CI: 44.7–55.4) FSWs reported that both of their biological parents were alive compared to a third (31.8%, 95% CI: 27.0–36.9) in Bobo-Dioulasso. Almost half of FSWs were foreigners, except in Bobo-Dioulasso where non-Burkina Faso nationals were 19.6% of participants (95% CI: 15.7–24.2).

*Socioprofessional Characteristics*. As shown in Table 2, earlier age of initiation to sex work was observed in Ouagadougou and Tenkodogo. The time spent in sex work varied across cities. In Ouagadougou, 40.9% (95% CI: 35.8–46.3) of FSWs had been doing this work for two years whereas, in Bobo-Dioulasso, 27.7% (95% CI: 23.1–32.9) had worked for two years. Those who had done sex work for two to five years were estimated to be 38.2% (95% CI: 34.2–44.6) in Ouagadougou and 33.0% (95% CI: 28.1–38.3) in Bobo-Dioulasso. Conversely, the majority of FSWs had been in sex work for less than two years in smaller cities as seen in Koudougou (61.0%, 95% CI: 51.7–69.6), Ouahigouya (73.3%, 95% CI: 65.1–80.2), and Tenkodogo (60.4%, 95% CI: 51.2–68.9).

*HIV Prevalence*. As shown in Table 3, among Ouagadougou, Koudougou, and Ouahigouya FSWs, RDS-weighted HIV prevalence was 13.5% (95% CI: 9.6–18.7), 13.3% (95% CI: 7.6–22.4), and 13.0% (95% CI: 7.6–21.3), respectively, as compared to 30.1% (95% CI: 25.5–35.1) in Bobo-Dioulasso. The lowest prevalence was observed in Tenkodogo with 4.4% (95% CI: 2.2–8.8).

### 3.2. Bivariate

As also shown in Table 3, in unweighted bivariate logistic regression analyses, the HIV prevalence was statistically significantly different across the cities (*p* = 0.001) and age groups within the same city. Among FSWs who were less than 25 years old, 9.3% (95% CI: 5.3–15.9) tested positive for HIV as compared to 31.8% (95% CI: 20.2–46.3) among those 30 years old and above in Ouagadougou (*p* = 0.001). Likewise, in all the other cities, FSWs aged 30 years and above were the most likely to test positive for HIV.

As shown in Table 4, in the bivariate analysis, FSWs who had symptoms of STIs in the past 12 months were 64% more likely to test positive for HIV compared to those who did not have symptoms (odds ratio [OR] = 1.64, 95% CI: 1.18–2.30).

### 3.3. Multivariate

As also shown in Table 4, the association between STI symptoms and HIV was not significant in the multivariate analysis (adjusted odds ratio [aOR] = 1.44, 95% CI: 0.98–2.13, *p* = 0.063). Our multivariate analysis showed that FSWs aged 30 years old and above were more likely to test positive for HIV (aOR = 7.84, 95% CI: 3.78–16.20) compared to those aged 18 to 24 years. Those married or cohabitating were also more likely to test positive for HIV than single FSWs (aOR = 2.43, 95% CI: 1.31–4.49). There was an association between divorced or widow status and HIV infection; however, it was not statistically significant in the multivariate analysis (aOR = 1.58, 95% CI: 0.99–2.51, *p* = 0.052).

Compared to FSWs who had between one and 14 clients per week, those who had more than 30 clients per week were less likely to test positive for HIV (aOR = 0.49, 95% CI: 0.27–0.87). The likelihood of testing positive for HIV was higher among those who had previously been pregnant (aOR = 5.24, 95% CI: 1.44–18.97), as well as those who reported a condom broke during sexual intercourse in the last 12 months (aOR = 1.78, 95% CI: 1.16–2.73).

## 4. Discussion

FSWs who lived in larger cities, were older, and engaged in condomless intercourse or became pregnant were more likely to be HIV positive in this sample. This data shows that, despite the continuous decline of HIV prevalence among the general population of reproductive age, dropping from 2.7% in 2003 to 0.9% in 2014, the prevalence among FSWs is still very high in the country.

In this study, the HIV prevalence was higher in the larger cities compared to the smaller cities which is concurrent with other West African settings. In a study conducted in Côte d'Ivoire in the following cities, Abidjan, Yamoussoukro, Gagnoa, and San Pedro, HIV prevalence was 17.5%. But in Abidjan, the largest city, it was 31.1% [29].

In this study, older FSW, aged 25 and older, had a higher prevalence of HIV, with those over 30 having the highest prevalence at 31.8% compared to younger FSW aged 24 and younger. HIV prevalence among FSWs remains five times higher than the prevalence in the same age group in the general population of reproductive age in Burkina Faso [19]. Some previous studies have shown that HIV is less prevalent among younger FSWs compared to older ones. Older FSWs may have greater accumulated vulnerability because they have engaged in sex work longer, potentially with more clients over time making them more likely to acquire HIV [30].

FSWs in Burkina Faso engaged in behavior increasing the likelihood of HIV transmission, including condomless sex with some clients [27]. In the study sample, condom breakage during sexual intercourse and history of previous pregnancies were associated with HIV infection among FSWs. Pregnancy increases vulnerability to HIV among FSWs. A pregnancy in the context of sex work assumes that the FSW engaged in condomless vaginal intercourse with clients or nonpaying partners, which can lead to HIV infection. In fact, FSWs who intend to conceive are more likely to have condomless sex and therefore more likely to contract HIV [31]. Although the design of this study could not establish whether HIV or condomless sexual intercourse occurred first, systematic and regular use of condoms remains a reliable method to prevent both HIV infection and unintended pregnancies during sex work [32, 33].

These results illustrate a need to strengthen HIV and STI prevention programs among FSWs in general. In particular, interventions focusing on older FSWs who have been working longer are needed to create a safe working environment. HIV response programs should reinforce HIV awareness and communication for behavior change strategies and access to preexposure prophylaxis among FSWs and their clients. These strategies should include access to condoms and education on correct utilization in order to prevent condom breakage which can lead to HIV transmission [27, 34, 35]. Previous studies indicate the importance of peer-based education. As seen in the Yerelon cohort in Bobo-Dioulasso, peer-based education intervention can result in positive changes in sexual behavior and low HIV incidence among FSWs [36]. This data helps us better understand the needs of FSW that are opportunities for improved future interventions.

## 5. Limitations

Our study has some limitations pertaining to the RDS method [31–34] specifically that the data across cities cannot be pooled because the networks, chains, and seeds are unique to each city. In addition, self-reported data are subject to inaccurate recall and social desirability bias. Despite these limitations, this study shows that the RDS method is feasible among FSWs in both large and small cities in Burkina Faso as it has been in other countries and settings.

## 6. Conclusions

In Burkina Faso, HIV prevention programs for vulnerable populations, namely, FSWs, are crucial, as HIV prevalence in West Africa is concentrated in these groups. With current interventions, we are still seeing high prevalence among this vulnerable population and we should take these results as an opportunity to change HIV prevention efforts towards FSWs. The results of this study suggest innovative, evidence-based HIV prevention interventions are needed especially in larger cities particularly among older FSWs because the prevalence is high. These interventions should benefit FSWs of all ages, especially older FSWs aged 25 and older in different locations throughout the country. Programs supporting FSWs could offer increased services to this population including increasing availability of STI testing, self-testing for HIV, mental health counselling and support, condom and lubricant distribution, and evaluation of FSWs as candidates for preexposure prophylaxis as it becomes available in Burkina Faso. Preexposure prophylaxis would provide FSWs an additional way to protect themselves from HIV and create a safer environment for them to work in. There has been previous success with peer-based education which could potentially be scaled up to a larger level and to more cities. The cost effectiveness of comprehensive HIV prevention programs for FSWs needs further evaluation to inform potential scale-up of these interventions in Burkina Faso.

## Figures and Tables

**Table 1:** Sociodemographic characteristics of female sex workers by city.

	Variables	Ouagadougou (*n* = 349)		Bobo-Dioulasso (*n* = 350)		Koudougou (*n* = 117)		Ouahigouya (*n* = 121)		Tenkodogo (*n* = 136)	
		*n* (% unadjusted)	RDS-adjusted% (95% CI)	*n* (% unadjusted)	RDS-adjusted% (95% CI)	*n* (% unadjusted)	RDS-adjusted% (95% CI)	*n* (% unadjusted)	RDS-adjusted% (95% CI)	*n* (% unadjusted)	RDS-adjusted% (95% CI)
Age (years)	18–24	203 (58.2)	63.6 (58.1–68.1)	101 (28.9)	32.1 (27.2–37.3)	47 (40.2)	32.2 (24.6–40.9)	59 (48.8)	44.9 (36.2–53.9)	86 (63.2)	65.5 (57.1–73.1)
	25–29	83 (23.8)	18.9 (15.4–23.0)	67 (19.1)	21.5 (17.3–26.4)	37 (31.6)	40.9 (31.8–50.7)	31 (25.6)	25.5 (18.5–34.2)	33 (24.3)	25.5 (18.7–33.7)
	30–55	63 (18.1)	17.5 (13.9–21.8)	182 (52.0)	46.5 (41.3–51.7)	33 (28.2)	26.8 (19.5–35.6)	31 (25.6)	29.6 (21.7–38.8)	17 (12.5)	9.0 (5.6–14.2)

Education	None	82 (23.8)	31.6 (26.5–37.2)	145 (41.5)	49.2 (43.8–54.5)	32 (27.4)	32.3 (24.0–41.9)	40 (33.1)	41.5 (32.6–51.0)	37 (27.2)	23.4 (17.2–30.9)
	Primary	131 (38.0)	35.7 (30.9–41.0)	130 (37.1)	34.9 (30.1–40.0)	47 (40.2)	40.7 (32.0–50.0)	28 (23.1)	21.3 (15.0–29.4)	48 (35.3)	33.0 (25.6–41.3)
	Secondary and above	132 (38.3)	32.7 (28.0–37.7)	75 (21.4)	15.9 (12.8–19.7)	38 (32.5)	27.1 (20.0–35.5)	53 (43.8)	37.2 (29.1–46.0)	51 (37.5)	43.6 (35.2–52.3)

Marital status	Single	220 (63.0)	55.7 (50.2–61.0)	156 (44.6)	51.9 (46.6–57.1)	81 (69.2)	72.0 (63.1–79.4)	83 (68.6)	66.8 (57.7–74.9)	103 (75.7)	74.0 (65.2–81.2)
	Married/cohabitating	33 (9.5)	8.7 (6.3–12.1)	43 (12.3)	12.5 (9.4–16.4)	11 (9.4)	5.4 (3.0–9.7)	13 (10.7)	10.3 (6.0–17.1)	6 (4.4)	8.4 (3.8–17.4)
	Divorced/separated/widowed	96 (27.5)	35.6 (30.4–41.2)	151 (43.1)	35.7 (30.9–40.7)	25 (21.4)	22.6 (15.7–31.4)	25 (20.7)	22.8 (15.9–31.6)	27 (19.9)	17.6 (12.2–24.7)

Occupation	Student/pupil	20 (5.8)	5.6 (3.6–8.6)	8 (2.3)	1.5 (0.7–3.0)	6 (5.1)	5.1 (2.3–11.1)	4 (3.3)	4.7 (1.8–11.9)	13 (9.6)	14.1 (8.4–22.7)
	Employed (public or private)	151 (43.5)	44.3 (39.1–49.6)	100 (28.6)	24.0 (20.0–28.5)	58 (49.6)	49.6 (40.5–58.7)	46 (38.0)	36.3 (28.2–45.3)	44 (32.4)	21.5 (16.0–28.3)
	Unemployed	176 (50.7)	50.1 (44.8–55.3)	242 (69.1)	74.5 (70.0–78.6)	53.0 (45.3)	45.3 (36.4–54.5)	71 (58.7)	59.0 (49.0–67.5)	79 (58.1)	64.4 (55.7–72.1)

Number of children	0	107 (30.7)	30.4 (25.8–35.4)	58 (16.6)	16.2 (12.7–20.4)	29 (25.2)	25.0 (18.0–34.0)	30 (29.1)	28.8 (20.9–38.3)	55 (40.7)	40.3 (32.4–48.8)
	1	157 (45.0)	44.9 (39.7–50.2)	103 (29.4)	29.1 (24.5–34.0)	48 (41.7)	41.3 (32.7–50.5)	42 (40.8)	40.5 (31.5–50.3)	48 (35.6)	36.1 (28.4–44.6)
	≥2	85 (24.4)	24.7 (20.4–29.5)	189 (54.0)	54.8 (49.5–59.9)	38 (33.0)	33.6 (25.5–42.8)	48 (35.6)	30.7 (22.5–40.3)	32 (23.7)	23.6 (17.2–31.5)

Parents alive	Both alive	174 (50.6)	50.0 (44.7–55.4)	105 (30.0)	31.8 (27–36.9)	47 (40.9)	39.1 (30.6–48.4)	59 (49.6)	47.1 (38.2–56.3)	71 (52.2)	59.8 (51.4–67.6)
	Only one alive	129 (37.5)	32.5 (27.9–37.5)	158 (45.1)	43.8 (38.6–49.0)	51 (44.3)	44.9 (35.9–54.2)	43 (36.1)	33.0 (25.1–41.9)	46 (33.8)	29.4 (22.5–37.4)
	None alive	41 (11.9)	17.4 (13.2–22.7)	87 (24.9)	24.5 (20.3–29.3)	17 (14.8)	16.0 (10.1–24.3)	17 (14.3)	19.9 (12.9–29.5)	19 (14.0)	10.8 (6.9–16.5)

Migrated into Burkina Faso	No	232 (67.1)	66.9 (61.7–71.7)	284 (81.1)	81.0 (76.5–84.8)	54 (47.4)	47.1 (38.1–56.3)	44 (43.1)	43.4 (34.1–53.2)	68 (54.0)	52.0 (41.8–58.6)
	Yes	114 (32.9)	42.7 (37.3–48.3)	66 (18.9)	19.6 (15.7–24.2)	62 (53.4)	51.3 (42.1–64.0)	61 (58.0)	53.7 (44.6–62.5)	67 (49.3)	47.9 (39.5–56.4)

**Table 2:** Socioprofessional and behavioral characteristics of female sex workers by city.

	Variables	Ouagadougou (*n* = 349)		Bobo-Dioulasso (*n* = 350)		Koudougou (*n* = 117)		Ouahigouya (*n* = 121)		Tenkodogo (*n* = 136)	
		*n* (% unadjusted)	RDS-adjusted% (95% CI)	*n* (% unadjusted)	RDS-adjusted% (95% CI)	*n* (% unadjusted)	RDS-adjusted% (95% CI)	*n* (% unadjusted)	RDS-adjusted% (95% CI)	*n* (% unadjusted)	RDS-adjusted% (95% CI)
Age at start of selling sex (years)	<20	226 (64.9)	64.8 (59.6–69.6)	173 (49.4)	48.5 (43.2–53.7)	44 (39.6)	39.7 (31.1–49.1)	38 (38.0)	37.7 (28.8–47.6)	81 (60.4)	60.3 (51.8–68.3)
	20–24	81.0 (23.3)	23.4 (19.2–28.1)	67 (19.1)	19.1 (15.3–23.6)	41 (36.9)	36.9 (28.5–46.3)	34 (34.0)	34.0 (25.4–43.8)	36 (26.9)	27.0 (20.1–35.2)
	≥25	41.0 (11.8)	11.9 (8.9–15.7)	110 (31.4)	32.4 (27.7–37.6)	26 (23.4)	23.3 (16.4–32.1)	28 (28.0)	28.3 (20.3–38.0)	17 (12.7)	12.7 (8.0–19.5)

Experience in sex work (years)	<1	59 (17.1)	17.0 (13.4–21.3)	35 (10.0)	09.7 (07.0–13.2)	35 (37.0)	30.1 (22.5–39.1)	52 (54.2)	53.9 (43.8–63.6)	62 (47.7)	47.6 (39.2–56.2)
	1–5	204 (59.0)	59.0 (53.7–64.1)	154 (44.0)	43.3 (38.2–48.5)	63 (55.3)	55.3 (46.0–64.1)	37 (38.5)	39.0 (29.7–49.1)	49 (37.7)	37.7 (29.8–46.4)
	≥6	83 (24.0)	24.0 (19.8–28.8)	161 (46.0)	47.0 (41.8–52.3)	16 (14.0)	14.6 (09.1–22.5)	7 (7.3)	07.1 (03.4–14.2)	19 (14.6)	14.6 (09.5–21.8)

Number of clients per week	1–14	214 (61.5)	61.8 (56.6–66.8)	146 (41.7)	42.2 (37.1–47.4)	73 (63.5)	63.5 (54.3–71.8)	50 (48.5)	48.5 (39.0–58.1)	82 (1.2)	61.2 (52.7–69.1)
	15–29	92 (26.4)	26.3 (21.9–31.2)	132 (37.7)	37.8 (32.9–43.0)	25 (21.7)	21.8 (15.2–30.4)	9 (8.7)	08.7 (04.6–15.9)	18 (13.4)	13.3 (08.5–20.1)
	≥30	42 (12.1)	11.9 (08.9–15.7)	72 (26.0)	20.0 (16.2–24.5)	17 (14.8)	14.7 (09.3–22.4)	44 (42.7)	42.8 (33.6–52.5)	34 (25.4)	25.5 (18.8–33.6)

Mean income per week (XOF, $US 1~500 XOF)	<15000	29 (8.3)	20.0 (14.6–26.8)	59 (16.9)	23.7 (19.0–29.1)	21 (17.9)	27 (18.6–37.4)	41 (35.3)	35.3 (27.1–44.6)	36 (27.1)	27.1 (20.1–35.4)
	15000–35000	147 (42.1)	35.3 (30.4–40.6)	178 (50.9)	47.9 (42.6–53.2)	35 (29.9)	29.5 (21.7–38.6)	43 (37.1)	37.1 (28.7–46.3)	76 (57.1)	57.1 (48.5–65.4)
	35001–99999	127 (36.4)	34.8 (29.7–40.1)	103 (29.4)	27.1 (22.7–31.9)	43 (36.8)	24.5 (18.1–32.3)	27 (23.3)	23.3 (16.4–32.0)	20 (15.0)	15.0 (9.9–22.3)
	≥100000	46 (13.2)	9.9 (7.4–13.1)	10 (2.8)	1.4 (0.7–2.5)	18 (15.4)	19.0 (12.3–28.2)	5 (4.3)	4.3 (1.8–10.1)	1 (0.8)	0.8 (0.1–5.3)

STI symptoms over the last 12 months	No	175 (50.3)	52 (45.0–55.5)	166 (47.8)	47.8 (42.5–53.0)	69 (60)	59.6 (50.3–68.2)	67 (65.0)	64.2 (54.5–73.0)	101 (75.9)	75.8 (67.7–82.3)
	Yes	172 (49.4)	49.5 (44.2–54.7)	181 (52.2)	52.2 (47.0–57.5)	46 (40)	40.4 (31.8–49.7)	34 (33.0)	33.9 (25.3–43.6)	32 (24.1)	24.2 (17.7–32.3)
	Don't know	01 (0.3)	0.3 (00–02.0)	—		—		02 (02.0)	00.9 (00.1–06.4)	—	

Ever got pregnant	No	74 (21.3)	21.0 (17.0–25.6)	29 (8.3)	07.9 (05.6–11.2)	22 (19.1)	18.8 (12.7–26.9)	20 (19.4)	19.1 (12.7–27.9)	44 (32.6)	32.2 (24.9–40.5)
	Yes	274 (78.7)	79.0 (74.4–83.0)	321 (91.7)	92.1 (88.8–94.4)	93 (89.0)	81.2 (73.1–87.3)	83 (86.0)	80.9 (72.1–87.3)	91 (67.4)	67.8 (59.5–75.1)

Ease of requesting client to wear a condom	Easy	238 (68.4)	68.4 (63.4–73.1)	246 (71.1)	71.0 (65.9–75.5)	83 (75.5)	75.2 (66.3–82.4)	96 (93.2)	93.4 (86.7–96.8)	124 (92.5)	92.6 (86.8–96.0)
	Not easy	110 (31.6)	31.6 (26.9–36.6)	100 (28.9)	29.0 (24.5–34.1)	27 (24.5)	24.8 (17.6–33.7)	7 (6.8)	06.6 (03.2–13.3)	10 (7.5)	07.4 (04.0–13.2)

Consistent condom use during the last 12 months	No	129 (37.7)	37.6 (32.6–42.8)	150 (43.1)	43.2 (38.0–48.5)	20 (17.9)	17.7 (11.7–25.9)	54 (56.8)	56.9 (46.7–66.5)	53 (48.0)	40.9 (32.7–49.5)
	Yes	213 (62.3)	62.4 (57.2–67.4)	198 (56.9)	56.8 (51.5–62.0)	92 (82.1)	82.3 (74.1–88.3)	41 (43.2)	43.1 (33.5–53.3)	77 (59.2)	59.1 (50.5–67.3)

Condom use during the last intercourse with new client	No	36 (17)	15 (07.7–14.3)	29 (8.6)	08.8 (06.2–12.4)	9 (7.9)	07.9 (04.2–14.5)	16 (15.8)	15.6 (09.8–24.1)	4 (3.0)	02.9 (01.1–07.6)
	Yes	301 (89.3)	89.5 (85.7–92.3)	309 (91.4)	91.2 (87.6–93.8)	105 (92.1)	92.1 (85.5–95.8)	85 (84.2)	84.4 (75.9–90.2)	131 (97.0)	97.1 (92.4–98.9)

Condom breakage over the last 12 months	No	128 (36.7)	36.6 (31.7–41.7)	134 (38.3)	37.8 (32.9–43.0)	47 (49)	32.8 (24.4–42.4)	34 (33.0)	40.5 (31.9–49.7)	70 (51.9)	51.4 (43.0–59.7)
	Yes	221 (63.3)	63.4 (58.3–68.3)	216 (61.7)	62.2 (57.0–67.1)	68 (59.1)	59.5 (50.3–68.1)	69 (67.0)	67.2 (57.6–75.6)	65 (48.1)	48.6 (40.3–57.0)

**Table 3:** HIV prevalence among female sex workers by city.

Variables		Ouagadougou			Bobo-Dioulasso			Koudougou			Ouahigouya			Tenkodogo		
	Age (years)	*n*	% unadjusted	RDS- adjusted% (95% CI)	*n*	% unadjusted	RDS- adjusted% (95% CI)	*n*	% unadjusted	RDS-adjusted% (95% CI)	*n*	% unadjusted	RDS-adjusted% (95% CI)	*n*	% unadjusted	RDS- adjusted% (95% CI)
HIV prevalence	18–24	203	5.4	9.3 (5.3–15.9)	101	4.0	3.8 (1.4–9.7)	46	6.5	9.2 (3.0–25.0)	51	5.9	6.1 (1.9–17.0)	85	3.5	2.6 (0.8–8.0)
	25–29	83	4.8	8.3 (3.2–20.0)	37	6.4	15.8 (8.9–26.4)	36	8.3	11.7 (3.8–30.5)	27	22.2	22.8 (10.5–42.7)	33	9.1	6.9 (2.2–19.8)
	30–55	63	20.6	31.8 (20.2–46.3)	182	51.6	50.4 (43.2–57.7)	33	15.2	20.6 (9.0–40.6)	25	16.0	16.5 (6.2–36.9)	17	11.8	9,0 (2.2–30.9)
	Total	349	8.0	13.5 (9.6–18.7)	350	31.1	30.1 (25.5–35.1)	115	9.6	13.3 (7.6–22.4)	103	12.6	13 (7.6–21.3)	135	5.9	4.4 (2.2–8.8)

**Table 4:** Factors associated with HIV positive status among female sex workers in five cities in Burkina Faso (*n* = 1052).

	Variables	*n* (% HIV positive)	Bivariate analysis			Multivariate analysis		
			OR	95% CI	*p*	aOR	95% CI	*p*
Sociodemographic characteristics	*Total*	1052 (16.1)	—	—	—	—	—	—
	*Age (years)*							
	18–24	486 (4.9)	1			1		
	25–29	246 (27.0)	2.37	1.33–4.21	*0.003*	1.76	0.90–3.45	*0.095*
	30–55	320 (36.9)	11.24	7.02–18.00	*0.001*	7.84	3.78–16.20	*0.001*
	*Education*							
	None	243 (7.0)	1			1		
	Primary	507 (13.2)	0.79	0.55–1.16	*0.242*	0.88	0.57–1.36	*0.585*
	Secondary and above	286 (29.0)	0.39	0.25–0.62	*0.001*	0.71	0.42–1.19	*0.199*
	*Marital status*							
	Single	630 (8.9)	1			1		
	Married/cohabitating	102 (27.5)	3.87	2.32–6.48	*0.001*	2.43	1.31–4.49	*0.005*
	Divorced/separated/widowed	320 (26.6)	3.71	2.56–3.37	*0.001*	1.58	0.99–2.51	*0.052*
	*Occupation*							
	Student/pupil	49 (0.0)	—			—	—	—
	Employed (public or private)	391 (15.9)	0.88	0.63–1.25	*0.488*	—	—	—
	Unemployed	610 (17.5)	1			—	—	—
	*Number of children*							
	0	279 (6.5)	1			1		
	1	398 (12.3)	2.03	1.16–3.58	*0.013*	0.82	0.40–1.65	*0.583*
	≥2	375 (27.2)	2.41	3.19–9.19	*0.001*	0.74	0.35–1.56	*0.440*
	*Parents alive*							
	One died	596 (19.3)	1			1		
	Both alive	447 (11.6)	0.55	0.38–0.78	*0.001*	1.25	0.81–1.94	*0.307*

Sex work-related characteristics	*Migrated into Burkina Faso*							
	No	682 (16.7)	1					
	Yes	365 (14.8)	0.86	0.61–1.23	*0.420*	—	—	—
	*Age at start of selling sex (years)*							
	<20	562 (11.0)	1			1		
	20–24	259 (15.1)	1.42	0.92–2.19	*0.104*	1.27	0.74–2.17	*0.376*
	≥25	222 (30.2)	3.48	2.36–5.14	*0.001*	1.06	0.59–1.90	*0.844*
	*Experience in sex work (years)*							
	Less than one year	243 (7.0)	1			1		
	1–5 years	507 (13.2)	2.02	1.16–3.52	*0.013*	1.33	0.70–2.53	*0.375*
	6 years and more	286 (29.0)	5.43	3.11–9.46	*0.001*	1.57	0.76–3.22	*0.223*
	*Number of clients per week*							
	1–14	565 (16.8)	1			1		
	15–29	276 (18.8)	1.14	0.79–1.66	*0.468*	0.91	0.59–1.41	*0.691*
	30 and more	209 (10.5)	0.58	0.35–0.95	*0.032*	0.49	0.27–0.87	*0.015*
	*STI symptoms over the last 12 months*							
	No	578 (13.0)	1			1		
	Yes	465 (19.8)	1.64	1.18–2.30	*0.003*	1.44	0.98–2.13	*0.063*
	*Ever got pregnant*							
	No	189 (2.1)	1			1		
	Yes	862 (19.1)	10.94	4.00–29.9	*0.001*	5.24	1.44–18.97	*0.012*
	*Ease of requesting client to wear a condom*							
	Easy	787 (15.5)	1					
	Difficult	254 (18.1)	1.20	0.83–1.75	*0.326*	—	—	—
	*Consistent condom use during the last 12 months*							
	No	406 (16,5)	1					
	Yes	621 (15.7)	0.95	0.67–1.33	*0.758*	—	—	—
	*Condom use during the last intercourse with new client*							
	No	94 (16.0)	1					
	Yes	931 (15.9)	0.99	0.57–1.77	*0.988*	—	—	—
	*Condom breakage over the last 12 months*							
	No	413 (11.4)	1			1		
	Yes	639 (19.1)	1.84	1.27–2.64	*0.001*	1.78	1.16–2.73	*0.007*
